# Consensus development and validation of entrustable professional activities for nephrology fellowship training in Saudi

**DOI:** 10.5116/ijme.6819.d7ce

**Published:** 2025-05-22

**Authors:** Manal E. Alotaibi, Faisal Basonbul, Sultan Al Dalbhi, Laila A. Alharbi, Amal M. Alkhotani, Hussam M. Alim, Fahd Almalki, Mohammed S. Samannodi, Hadeel A. Khadawardi, Ahmad A. Imam, Yosra A. Turkistani, Abdullah Tawakul, Adeeb A. Bulkhi, Mohammad S. Dairi, Rania Zaini, Hani M. Almoallim

**Affiliations:** 1 Department of Medicine, Medical College, Umm Al-Qura University (UQU), Makkah, Saudi Arabia; 2 King Faisal Specialist Hospital and Research Center Riyadh (KFSHRC), Saudi Arabia; 3 Department of Nephrology and Kidney Transplantation, Prince Sultan Military Medical City (PSMMC), Riyadh Saudi Arabia; 4 Department of Community Medicine, Medical College, Umm Al-Qura University Makkah, Saudi Arabia

**Keywords:** Entrustable professional activities, EPAs, nephrology, fellowship, medical education, competency by design, training programs, Saudi Arabia

## Abstract

**Objectives:**

This study aimed to develop and validate entrustable professional activities (EPAs) for the nephrology fellowship program in Saudi Arabia.

**Methods:**

This study utilized a two-round modified Delphi design involving nephrology consultants across Saudi Arabia. The initial list of 39 EPAs was created through a literature review and expert input, followed by a piloting process that refined these activities based on feedback. A target sample of 26 nephrology consultants was invited, achieving an 80% response rate in the first round with 21 participants. Descriptive statistics, including means and percentages, summarized demographic characteristics and group responses. Participants rated the relevance of each EPA using a 5-point Likert scale. Consensus was defined as at least 75% agreement among participants, which guided the refinement of EPAs across the Delphi rounds. The study was approved by the institutional review board at Umm Al-Qura University, Makkah, Saudi Arabia.

**Results:**

In the first round, consensus was achieved for 34 EPAs, while 5 were excluded for lack of relevance and 6 were modified. The second round confirmed full consensus on the revised 34 EPAs, with an 81% response rate among the 21 experts.

**Conclusions:**

This study successfully developed and validated EPAs for the nephrology fellowship program in Saudi Arabia. Implementing these EPAs is expected to enhance training, assessment, and clinical competence for nephrology fellows. Future studies should explore the long-term impact of these EPAs on training outcomes and consider adapting them for other specialties.

## Introduction

EPAs are defined as responsibilities or tasks that can be entrusted to trainees for unsupervised practice once they have attained sufficient specific competencies.^1^^, ^^2^ First published in 2005.^3^ EPAs have since been supported by workshops, courses, and conferences aimed at helping faculty develop programs and assessment procedures.^1^^, ^^4^^, ^^5^ However, there remains a significant need for clarification and effective implementation of EPAs.^4^ Well-written EPAs provide a natural framework for establishing training objectives, requiring comprehensive descriptions that justify each component1, ^4^ importantly, EPAs are not substitutes for competencies; they translate competencies into day-to-day clinical tasks.^4^^, ^^6^ Competencies describe the traits of a physician, while EPAs delineate the specific work.^1^^, ^^6^ Several published EPAs exist for nephrology postgraduate training programs, such as those from Canada and the United States.^2^^, ^^7^^-^^11^ However, adopting EPAs from other cultures can be challenging due to differences in local culture, patient values, and healthcare systems. Institutions worldwide that have adopted EPAs have developed and validated their own based on specific needs.^2^ The Canadian Medical Education Directives for Specialists (CanMEDS) framework^12^^,^^13^ widely used in postgraduate training, outlines physician competencies that extend beyond medical expertise, emphasizing roles that meet societal needs. In response, the Saudi Commission for Health Specialties (SCFHS) is integrating the CanMEDS framework into the core curriculum for all training programs, including the nephrology fellowship.^14^^,^^15^ For example, educators may struggle with defining the practical applications of these competencies and ensuring consistent assessment across diverse clinical settings. EPAs, in contrast, outline the essential tasks that a newly graduated nephrologist must perform independently, thus advancing competency-based medical education (CBME) by enabling specific clinical tasks to be entrusted to trainees once they are proficient.^2^^, ^^12^^, ^^16^^, ^^17^ The aim of this study is to develop and validate end-of-training EPAs for nephrology fellowship training in Saudi Arabia. This study is part of a larger initiative aimed at developing and validating EPAs across various internal medicine specialties in the country, building on previously published work that established foundational EPAs in related fields.^18^

## Methods

The study consisted of two phases: the first phase aimed to develop a preliminary list of EPAs for the nephrology fellowship program based on a comprehensive literature review and expert opinions. The second phase sought to establish consensus on the suggested EPA list through two rounds of Delphi techniques with nephrology experts. The study was approved by the institutional review board at Umm Al-Qura University, Makkah, Saudi Arabia.

### Study population and sampling

The study population included nephrology consultants in Saudi Arabia, each with over two years of experience in the field and involvement in postgraduate training programs. Consultants were selected through purposeful sampling to ensure geographic balance and subspecialty expertise across regions. Written informed consent was obtained from all participants.

### Demographic characteristics

Twenty-six nephrology consultants were invited to participate in the modified Delphi technique survey, achieving an 80% response rate with 21 participants. Most participants were male, constituting 85.7% of the group. In terms of nationality, 76% identified as Saudi. The age distribution revealed that most participants (71%) were between 35 and 44 years old. Regarding leadership roles, approximately 43% of the participants held positions within nephrology fellowship programs. Furthermore, regional representation included participants from Riyadh (43%), Jeddah (24%), and Makkah, Abha, and Taif, each contributing 10% to the cohort. (see Table 1).

### Preliminary phase

A preliminary set of EPAs was developed by three nephrologists and two medical educators, who conducted a thorough literature review of international EPAs and frameworks, including the SCFHS competency training framework for nephrology.^2^^,^^7^^-^^9^^,^^11^^,^^14^^, ^^17^^, ^^19^^-^^22^ This set was organized into four categories: clinical assessment, clinical management, procedures, and transferable skills. A pilot survey was then conducted to gather feedback on the clarity and relevance of the EPAs, leading to their refinement based on panelists’ qualitative comments. Specific modifications were made in response to the feedback, and additional demographic questions were included to enhance data collection.

### Second phase

The second phase involved two rounds of the Delphi technique conducted via online surveys. The first round included demographic data and the preliminary list of EPAs, where panelists rated the representativeness and relevance of each EPA on a 5-point Likert scale (1 = not important, 5 = extremely important). Panelists could also suggest changes or introduce new EPAs. The consensus criteria for retaining EPAs included an average rating of 4 or higher and at least 80% of panelists rating the EPA as 4 or 5. The mean and standard deviation were computed for each EPA, and modifications were made based on qualitative feedback.

In the second round, the same expert panel reviewed the modified list, using the same rating scale and providing feedback on the applied changes. The analysis of ratings and comments followed the same framework as the first round, ensuring consistency in evaluation.

### Internal consistency of Delphi rounds

Descriptive statistics summarized participants' demographic characteristics and group responses across all rounds. A target response rate of 80% was set. Cronbach’s alpha was calculated to assess the internal consistency of the ratings, indicating reliability in the assessments. Additionally, standard deviation (SD) was employed to evaluate the consensus among panelists regarding the mean values for each EPA. Statistical procedures were reported, including the presentation of results with means and standard deviations.

## Results

### Preliminary phase

In the preliminary phase, an initial list of 39 EPAs was developed for the nephrology fellowship program in Saudi Arabia. A draft Delphi study questionnaire was created and piloted among five nephrologists. The pilot results highlighted specific concerns about the clarity and relevance of some EPAs,

particularly regarding their applicability in clinical practice. In response, modifications were made to the wording of several EPAs to enhance clarity, and demographic questions were added to the final questionnaire to gather more comprehensive feedback from participants.

**Table 1.** Demographic characteristic of the participants in the first round of modified Delphi technique

Demographic characteristics		
Gender		N (%)
	Male	18 (85.7)
	Female	3 (14.2)
Nationality		
	Saudi	16 (76.1)
	Non-Saudi	5 (23.8)
Age		
	35-44	15 (71.4)
	45-54	4 (19)
	>55	2 (9.5)
Years of experience as Nephrologist		
	<5	5 (23.8)
	5-10	10 (47.6)
	>10	6 (28.5)
Years of experience in training programs		
	<2	4 (19)
	2-5	10 (47.6)
	6-10	4 (19)
	>10	3 (14.2)
Status in graduate training		
	PD	5 (23.8)
	Co-PD	2 (9.5)
	Former PD	2 (9.5)
	trainer	7 (33.3)
	Others	5 (23.8)
City of practice		
	Jeddah	5 (23.8)
	Makkah	2 (9.5)
	Riyadh	9 (42.8)
	Abha	2 (9.5)
	Dammam	1 (4.7)
	Taif	2 (9.5)
Affiliation		
	University (academic- governmental)	15 (71.4)
	University (academic- private)	3 (14.2)
	Health cluster / medical city	3 (14.2)

### First round of Delphi technique

In the first round of the Delphi technique, the 39 suggested EPAs were evaluated. Consensus was defined as achieving an average mean score of at least 4.0 and a rating of 4 or higher by over 80% of panel members. A total of 34 EPAs achieved this consensus. Based on expert feedback, which primarily highlighted concerns about redundancy and the feasibility of implementation, five EPAs were deleted. Additionally, the wording of six others was modified to improve clarity and applicability. This process resulted in a refined list of 34 pre-final EPAs (see Table 2).

### Second round of Delphi technique

The second round involved the same participants reviewing the modified, pre-final list of 34 EPAs. Of the 26 invited experts, 21 (81%) completed this round. Consensus was achieved for all 34 EPAs, each obtaining an average mean score of 4.0 or higher and over 80% agreement on their importance and relevance. Panelists evaluated the modifications made in the first round; 55% (11 out of 20 respondents) agreed with the amendments and deletions, while 45% disagreed. This disagreement was discussed among the panelists, highlighting the diverse perspectives on the issue. Further refinements were made to address the concerns raised, ultimately leading to a final consensus on the 34 end-of-training EPAs for the nephrology fellowship training program in Saudi Arabia (see Table 3).

### Internal consistency of Delphi rounds

The internal consistency of the modified Delphi rounds was assessed using Cronbach’s alpha, yielding values of α = 0.91 for the first round and α = 0.97 for the second round (see Table 2). Descriptive statistics, including means and standard deviations, were calculated for each EPA.

## Discussion

This study aimed to develop a consensus-based set of EPAs for Saudi Arabia’s nephrology fellowship programs.^2^^,^^19^^,^^23^ These EPAs are designed to enhance the training, assessment, and clinical skills of nephrology fellows, ultimately improving patient care in Saudi Arabia. To meet international standards while addressing local healthcare needs, it is essential to incorporate these EPAs into fellowship programs and evaluate their impact on trainee performance and patient outcomes. The development process followed established guidelines for creating effective EPAs.^5^ The initial list of EPAs and the number of nephrology consultants involved in the Delphi process were based on expert recommendations.^2^^,^^15^^,^^19^ Feedback from the panel of nephrologists was carefully reviewed, leading to necessary adjustments during the second round of the Delphi technique. This iterative process allowed the study to reach a consensus, resulting in a final list of 34 EPAs, consistent with literature suggestions.^5^^, ^^6^

The initiative is part of a broader institutional project to develop an EPA framework for Internal Medicine Specialized fellowship programs, promoting standardization in training. By providing a structured approach, these EPAs ensure that trainees acquire consistent skills and knowledge, leading to improved patient outcomes.^2^^,^^19^ Furthermore, the development of the EPAs framework supports the implementation of Competency-Based Medical Education (CBME) in nephrology training, allowing for focused and individualized assessments.^12^^,^^20^^,^^21^ EPAs outline the essential tasks that graduating nephrologists must perform independently in clinical practice.^6^ They encompass multiple competencies, allowing for varied approaches to achieving mastery. EPAs translate competencies into practical tasks, emphasizing hands-on experience to facilitate the transition from training to autonomous practice.^24^ Therefore, they should be viewed as part of a dynamic curriculum process, with regular reviews to align with evolving trends in nephrology healthcare practice.^5^ The alignment of EPAs with the Saudi Commission for Health Specialties (SCFHS) development and quality improvement initiatives is crucial. By identifying essential skills and knowledge required for nephrologists, programs can target areas for improvement, ensuring that trainees meet the evolving needs of patients.^17^^, ^^21^^, ^^25^ Feedback from nephrology consultants during the Delphi process led to adjustments in the EPAs.

**Table 2.** The final set of end-of-training entrustable professional activities (EPAs) for Nephrology fellowship training program in Saudi Arabia

EPA		MeanRound 1	SD	MeanRound 2	SD
A. Clinical assessment					
	A1. Obtaining comprehensive history and performing physical examinations in patients with renal presentations.	4.9	0.2	5	0
	A2. Composing diagnostic approach and treatment plans for patients with renal diseases.	4.7	0	5	0
	A3. Applying basic medical knowledge in daily medical practice (including anatomy, physiology, immunology, pathology, genetics and laboratory medicine).	4.6	0.7	4.9	0.3
	A4. Demonstrating expertise in the indications for and interpretation of diagnostic tests relevant to the evaluation of patients with suspected or established renal diseases.	4.4	0.6	4.761	0.4
	A5. Interpreting laboratory results or data related to the administration and/or contraindication of immunomodulatory therapy.	4.7	0.6	4.76	0.4
	A6. Demonstrating expertise in the indications for and interpretation of imaging studies relevant to the evaluation of patients with suspected or established renal diseases.	4.47	0.7	4.71	0.5
	A7. Recognizing rare renal disease presentations.	3.9	0.9		
B. Clinical Management					
	B1. Providing initial assessment and plan for investigation and management for patients with acute kidney disease or chronic kidney disease.	4.9	0.3	5	0
	B2. Composing initial assessment and plan for investigation and management for patients with complex fluid and electrolyte abnormalities.	4.9	0.2	4.9	0.3
	B3. Assessing and managing patients with hematuria and or proteinuria.	5	0	4.85	0.4
	B4. Triaging and proposing initial management of patients with emergency renal conditions.	4.8	0.4	4.95	0.2
	B5. Prescribing with proper adjustments of all modalities of renal replacement therapy.	4.8	0.4	4.85	0.4
	B6. Providing assessment and management plans for patients with acute or chronic complications of all modalities of renal replacement therapy.	4.7	0.4	4.9	0.3
	B7. Facilitating patients’ transition to an ESRD treatment modality, or to end of life care.	4.6	0.5	4.76	0.5
	B8. Applying longitudinal management for patients receiving chronic dialysis.	4.6	0.5	4.66	0.6
	B9. Providing pre- and post-operative care for renal transplant recipients with complicated and uncomplicated courses.	4.5	0.7	4.61	0.6
	B10. Providing assessment and management for patients with common complications of renal transplantation.	4.6	0.6	4.57	0.7
	B11. Assessing the suitability of deceased donors and potential living donors for kidney transplantation	4.4	0.9258201		
	B12. Assessing the eligibility of patients with renal disease for kidney transplantation.	4.5	0.7	4.8	0.4
	B13. Monitoring patients receiving immune modulating therapy and managing complications.	4.7	0.5	4.85	0.4
	B14. Integrating knowledge of the effects of pregnancy, pregnancy outcomes, renal disease, and its treatments in the care of women with renal disease.	4.7	0.4	4.8	0.4
	B15. Applying guidelines, evidence-based literature, and/or consensus treatment plans to the care of patients.	4.6	0.5	4.85	0.4
C. Procedure					
	C1. Performing and interpreting the results the urine microscopy.	4.6	0.9	4.6	0.6
	C2. Performing the placement of hemodialysis catheters and the removal of tunneled, cuffed hemodialysis catheters.	4.3	0.9		
	C3. Performing native and transplant kidney biopsy.	3.4			
D. Transferable skills					
	D1. Counselling patients and/or families regarding diagnosis and treatment plans for renal diseases.	4.7	0.5	4.95	0.2
	D2. Implementing the principles of quality assurance and patient safety.	4.3	0.7	4.76	0.5
	D3. Developing a personal learning plan for future practice and ongoing professional development.	4.6	0.6	4.57	0.7
	D4. Participating in and/or leading educational or administrative activities.	4.3	0.7	4.61	0.6
	D5. Delivering scholarly teaching to a variety of audiences, including peers, junior trainees and/or other health professionals.	4.3	0.7	4.52	0.7
	D6. Completing written documentation for patient care.	4.5	0.5	4.76	0.7
	D7. Managing a longitudinal clinic.	4.6	0.6	4.61	0.7
	D8. Working with the interprofessional team to coordinate the care of patients with renal diseases.	4.6	0.6	4.71	0.7
	D9. Monitoring one’s own practice and performance.	4.4	0.7	4.8	0.4
	D10. Critiquing and appraising current renal literatures.	4.5	0.6	4.47	0.7
	D11. Demonstrating professional consultancy skills utilizing resources and considering other specialties.	4.5	0.6	4.61	0.7
	D12. Promoting health in response to society needs.	4.1	0.9		
	D13. Providing/recommending appropriate referrals to other health care providers necessary for adjunctive evaluation and/or management.	4.6	0.5	4.71	0.5
	D14. Providing consultative care for patients with known renal disease admitted with other medical or surgical problems.	4.6	0.5	4.76	0.5

EPA= Entrustable Professional Activities; SD= Standard Deviation; ESKD= End Stage Kidney Disease; The EPAs in red color were removed.

**Table 3.** Final end-of-training entrustable professional activities (EPAs) list

Domain	Saudi EPAs
Clinical Assessment	1. Obtaining comprehensive history and performing physical examinations in patients with renal presentations.
	2. Composing diagnostic approach and treatment plans for patients with AKI, proteinuria, hematuria.
	3. Applying basic medical knowledge in daily medical practice, including anatomy, immunology, pathology, genetics of the most common inherited kidney abnormalities, and laboratory medicine.
	4. Demonstrating expertise in the indications for and interpretation of diagnostic tests relevant to the evaluation of patients with suspected or established renal diseases.
	5. Interpreting laboratory results or data related to the administration and/or contraindication of immunomodulatory therapy, prophylaxis and vaccination.
	6. Demonstrating expertise in the indications for and interpretation of imaging studies relevant to the evaluation of patients with suspected or established renal diseases.
Clinical Management	1. Providing initial assessment and plan for investigation and management for patients with acute kidney disease or chronic kidney disease.
	2. Composing initial assessment and plan for investigation and management for patients with complex fluid and electrolyte abnormalities
	3. Assessing and managing patients with hematuria and or proteinuria.
	4. Triaging and proposing initial management of patients with emergency renal conditions.
	5. Prescribing with proper adjustments of all modalities of renal replacement therapy.
	6. Providing assessment and management plans for patients with acute or chronic complications of all modalities of renal replacement therapy.
	7. Facilitating patients’ transition to an ESRD treatment modality, or to end of life care.
	8. Applying longitudinal management for patients receiving chronic dialysis.
	9. Providing pre- and post-operative care for renal transplant recipients with complicated and uncomplicated courses.
	10. Providing assessment and management for patients with common complications of renal transplantation.
	11. Assessing the eligibility of patients with renal disease for kidney transplantation.
	12. Monitoring patients receiving immune modulating therapy and managing complications.
	13. Integrating knowledge of the effects of pregnancy, pregnancy outcomes, renal disease, and its treatments in the care of women with renal disease.
	14. Applying guidelines, evidence-based literature, and/or consensus treatment plans to the care of patients.
Procedure	1. Performing and interpreting the results the urine microscopy.
Transferable skills	1. Counselling patients and/or families regarding diagnosis and treatment plans for renal diseases.
	2. Implementing the principles of quality assurance and patient safety.
	3. Developing a personal learning plan for future practice and ongoing professional development.
	4. Participating in and/or leading educational or administrative activities.
	5. Delivering scholarly teaching to a variety of audiences, including peers, junior trainees and/or other health professionals.
	6. Completing written documentation for patient care.
	7. Managing a long-term structured outpatient Nephrology clinic.
	8. Working with the interprofessional team to coordinate the care of patients with renal diseases.
	9. Monitoring one’s own practice and performance.
	10. Critiquing and appraising current renal literature or policies/protocols, such as allocation policies or donor assessment protocols.
	11. Demonstrating professional consultancy skills utilizing resources and considering other specialities.
	12. Providing/recommending appropriate referrals to other health care providers necessary for adjunctive evaluation and/or management
	13. Providing consultative care for patients with known renal disease admitted with other medical or surgical problems in a concise and clear manner.

For example, EPA A3 was modified to encompass a broader scope of "Applying basic medical knowledge in daily practice," reflecting the necessity for comprehensive training. Some EPAs, such as EPA B11, 'Assessing the suitability of deceased donors and potential living donors for kidney transplantation,' were excluded due to their specificity to transplant nephrologists, indicating the need for further training or dedicated fellowships. However, the importance of transplantation was emphasized, as fellows should manage pre- and post-operative care for renal transplant recipients and assess eligibility for kidney transplantation, as demonstrated in EPAs B9, 10, and 12.The exclusion of procedural skills, such as HD catheter placement (EPA C2) and kidney biopsies (EPA C3), sparked significant debate, particularly with nearly 45% of respondents disagreeing with these decisions in the second round. This highlights the ongoing discussions about nephrologists' responsibilities in their training.^20^^,^^22^^,^^26^^, ^^27^ While international standards from organizations like the American Society of Nephrology (ASN)^22^^, ^^28^ and Canadian EPAs,^8^ recognize certain procedures as essential to nephrology training, the common practice in Saudi hospitals often involves interventional radiologists performing these tasks. This situation complicates the discussion around the EPAs, as some important procedures, such as HD catheter insertion, are left out of the fellowship training framework. Despite this omission, gaining hands-on experience in these areas remains vital for fellows, helping them manage complications effectively and provide comprehensive care to dialysis patients. For example, learning how to insert HD catheters is a key part of nephrology training and fits well with guidelines for managing vascular access.^17^^, ^^22^^, ^^28^ Similarly, developing skills in native or transplant kidney biopsies is important for accurately diagnosing and treating patients.^26^^, ^^27^ This ongoing debate highlights the need to find a balance between global standards and local practices, ensuring that nephrology fellows are well-prepared to handle these critical procedures. We noted a 45% disagreement regarding the exclusion of certain EPAs. This analysis helped us understand the diverse perspectives on the issue. Ultimately, we decided not to make certain EPAs mandatory, believing that it's important for nephrology fellows to develop these essential skills at their own pace. This approach encourages engagement and motivation, allowing them to effectively tackle challenges in dialysis and nephrology care. In summary, our final decision balanced the need for skill development with the autonomy of individual learning.

The study has several limitations. Firstly, generalizability may be limited; while a diverse panel of nephrology experts from Saudi Arabia contributed, the findings might not be applicable to other regions. Future research should explore the generalizability of these EPAs. Additionally, the potential for expert bias exists, as the Delphi technique relies heavily on expert opinions. To mitigate this, future studies could incorporate broader methodologies, such as systematic literature reviews or focus groups. In terms of future research, concrete next steps could include longitudinal studies aimed at tracking the effectiveness of these EPAs in improving patient outcomes and the quality of fellowship training. Specific suggestions for implementing EPAs in Saudi fellowship programs include faculty training workshops, pilot programs, and establishing feedback loops for ongoing revisions. By integrating global standards while respecting local healthcare constraints, Saudi fellowship programs can enhance their training frameworks effectively.

## Conclusions

This study successfully established and validated EPAs for the Saudi nephrology fellowship training program, marking a meaningful progression in regional medical education. These EPAs aim to elevate the training, assessment, and clinical abilities of nephrology fellows in Saudi Arabia. To ensure they are in line with international standards while addressing the specific needs of local healthcare, it is vital to integrate these EPAs into fellowship programs and assess their effects on both trainee performance and patient care outcomes. Future research should prioritize validating this framework within the nephrology community, gathering insights from both educators and trainees to continuously refine and improve the EPAs. This ongoing feedback loop will be essential for adapting the EPAs to meet the dynamic challenges of nephrology practice in Saudi Arabia.

### Acknowledgements

The authors sincerely appreciate and extend their gratitude to the nephrology consultants who attended and participated in the modified Delphi rounds.

### Conflict of Interest

The author declares that there is no conflict of interest.
